# Botulinum Neurotoxin A Injected Ipsilaterally or Contralaterally into the Striatum in the Rat 6-OHDA Model of Unilateral Parkinson’s Disease Differently Affects Behavior

**DOI:** 10.3389/fnbeh.2017.00119

**Published:** 2017-06-21

**Authors:** Veronica A. Antipova, Carsten Holzmann, Oliver Schmitt, Andreas Wree, Alexander Hawlitschka

**Affiliations:** ^1^Institute of Anatomy, Rostock University Medical CenterRostock, Germany; ^2^Institute of Macroscopic and Clinical Anatomy, Medical University of GrazGraz, Austria; ^3^Institute of Medical Genetics, Rostock University Medical CenterRostock, Germany

**Keywords:** botulinum toxins, hemiparkinsonism, behavior, Wistar rats, striatum

## Abstract

Parkinson’s disease (PD) is one of the most frequent neurodegenerative disorders. The loss of dopaminergic neurons in the substantia nigra leads to a disinhibition of cholinergic interneurons in the striatum. Pharmacotherapeutical strategies of PD-related hypercholinism have numerous adverse side effects. We previously showed that ipsilateral intrastriatal injections of 1 ng in unilaterally 6-hydroxydopamine (6-OHDA)-lesioned rats inhibit apomorphine-induced rotation behavior significantly up to 6 months. In this study, we extended the behavioral testing of ipsilateral botulinum neurotoxin A (BoNT-A)-injection and additionally investigated the impact of intrastriatal BoNT-A-injections contralateral to the 6-OHDA-lesioned hemisphere on the basal ganglia circuity and motor functions. We hypothesized that the interhemispheric differences of acetylcholine (ACh) concentration seen in unilateral hemi-PD should be differentially and temporally influenced by the ipsilateral or contralateral injection of BoNT-A. Hemi-PD rats were injected with 1 ng BoNT-A or vehicle substance into either the ipsilateral or contralateral striatum 6 weeks after 6-OHDA-lesion and various behaviors were tested. In hemi-PD rats intrastriatal ipsilateral BoNT-A-injections significantly reduced apomorphine-induced rotations and increased amphetamine-induced rotations, but showed no significant improvement of forelimb usage and akinesia, lateralized sensorimotor integration and also no effect on spontaneous locomotor activity. However, intrastriatal BoNT-A-injections contralateral to the lesion led to a significant increase of the apomorphine-induced turning rate only 2 weeks after the treatment. The apomorphine-induced rotation rate decreases thereafter to a value below the initial rotation rate. Amphetamine-induced rotations were not significantly changed after BoNT-A-application in comparison to sham-treated animals. Forelimb usage was temporally improved by contralateral BoNT-A-injection at 2 weeks after BoNT-A. Akinesia and lateralized sensorimotor integration were also improved, but contralateral BoNT-A-injection had no significant effect on spontaneous locomotor activity. These long-ranging and different effects suggest that intrastriatally applied BoNT-A acts not only as an inhibitor of ACh release but also has long-lasting impact on transmitter expression and thereby on the basal ganglia circuitry. Evaluation of changes of transmitter receptors is subject of ongoing studies of our group.

## Introduction

Parkinson’s disease (PD) is one of the most prevalent debilitating chronic progressive neurodegenerative movement disorders and mainly caused by degeneration of dopaminergic neurons especially in the substantia nigra pars compacta (SNC; Hornykiewicz and Kish, 1987; Braak et al., 2004). This results in a deficit of striatal dopamine (DA) that leads to the impairment in cortico-striatal-thalamo-cortical or nigrostriatal pathways (Bagga et al., 2016; Inan et al., 2016; Kim et al., 2016) and is responsible for the major motor symptoms of PD, including muscular rigidity, resting tremor, bradykinesia and postural instability (Dauer and Przedborski, 2003; Kortekaas et al., 2005; Ren et al., 2016), and non-motor disturbances (Bargiotas and Konitsiotis, 2013).

In the striatum the decrease of DA is followed by an increase in the concentration of acetylcholine (ACh) released from disinhibited tonically active cholinergic striatal interneurons (Gerfen, 1994; Day et al., 2006; Pisani et al., 2007; Obeso et al., 2008b). Therefore, one possible therapeutic approach in PD is using anti-cholinergic drugs (Klockgether, 2003; Horstink et al., 2006). However, systemic application of anti-cholinergics has some peripheral and central side effects (Clarke, 2002; Fernandez, 2012; Connolly and Lang, 2014). To avoid these undesirable effects connected with systematic administration of anti-cholinergic drugs, we tested a local anti-cholinergic treatment by injecting botulinum neurotoxin A (BoNT-A) directly into the caudate putamen (CPu; Wree et al., 2011; Holzmann et al., 2012; Antipova et al., 2013; Hawlitschka et al., 2013; Mehlan et al., 2016) as a possible therapeutic option in experimental PD-model. In hemi-PD rats established by unilateral injection of 6-hydroxydopamine (6-OHDA) into the medial forebrain bundle (MFB; Ungerstedt, 1968; Ungerstedt and Arbuthnott, 1970; Meredith et al., 2008), intraperitoneally applied atropine antagonized profound PD-typical akinesia (Schallert et al., 1978) and in combination with L-DOPA suppressed pathological circling of hemi-PD rats (Schallert et al., 1979). In our previous studies (Wree et al., 2011) application of BoNT-A into the CPu ipsilateral to the dopaminergic depletion caused a long-term abolition of the pathological apomorphine-induced rotations. We hypothesized that due to the BoNT-A-application into the hypo-dopaminergic and hyper-cholinergic striatum of hemi-PD rats the cholinergic transmission is blocked thus leading to reduced pathological compensatory effects in this model.

In order to test our hypothesis that intrastriatal BoNT-A-application interferes with the 6-OHDA-induced hypercholinism (DeBoer et al., 1993) and/or the D_2_-receptor upregulation (Creese et al., 1977) in the CPu, we injected in the present study the “effective” dose of BoNT-A (Wree et al., 2011) either into the CPu ipsilateral or contralateral to the dopaminergic deprivation. Here we show that at least some of the behavioral effects seen after ipsilateral BoNT-A-applications in hemi-PD rats should be reversed or even changed to the opposite by contralateral BoNT-A-injection in hemi-PD rats.

## Materials and Methods

### Animals

Adult male Wistar rats, purchased at Charles River WIGA (Sulzfeld, Germany) and weighing 290–310 g at the time of first surgery were used. Rats were housed in standard cages at 22 ± 2°C under 12 h light/12 h dark cycle with free access of standard food and water. At the end of the experiments mean body weights were as follows: ipsilateral BoNT-A group 559.4 g ± 20.0; ipsilateral sham group 558.4 g ± 23.71; contralateral BoNT-A group 558.5 g ± 16.16; contralateral sham group 555.3 g ± 17.52.

### Stereotactic Intervention of Animal Groups

All animals got a 6-OHDA-injection into the MFB of the right hemisphere (hemi-PD), and the successfully lesioned rats were divided into two groups: (1) 6-OHDA-lesioned animals receiving BoNT-A into the CPu of the right hemisphere (ipsilateral BoNT-A group); and (2) 6-OHDA-lesioned animals receiving BoNT-A into the CPu of the left hemisphere (contralateral BoNT-A group), each group added with respectively sham-injected animals. All groups were created by the outcome of the apomorphine-induced rotational behavior after 6-OHDA in that the means of the BoNT-A-injected and the vehicle-injected rats inside (1) and (2) did not differ significantly. All experiments were approved by the State Animal Research Committee of Mecklenburg-Western Pomerania (LALLF M-V/TSD/7221.3-1.1-003/13).

**Figure 1:**
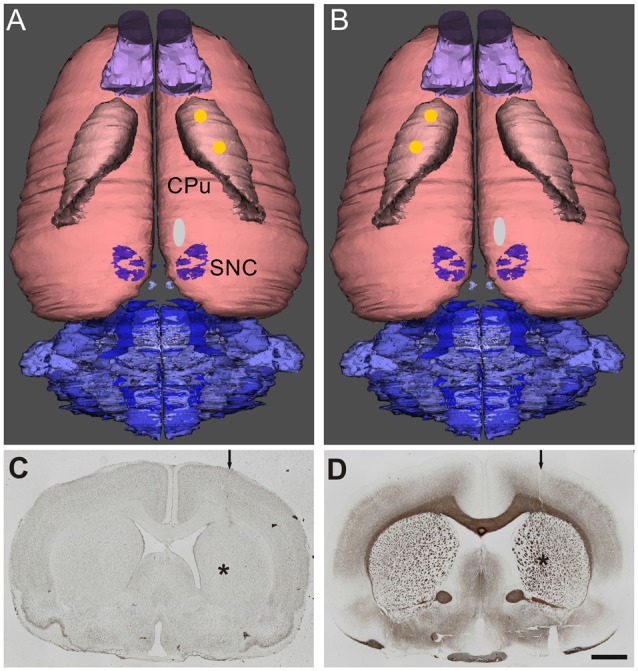
Dorsal view of reconstructed rat brains. The location of the right side 6-hydroxydopamine (6-OHDA)-injection site in the medial forebrain bundle (MFB; white oval) anterior to the substantia nigra pars compacta (SNC) and the two injection sites of botulinum neurotoxin A (BoNT-A) or vehicle (yellow spots) in the caudate putamen (CPu) ipsilateral **(A)** and contralateral **(B)** to 6-OHDA are indicated. Adjacent 20 μm coronal sections of a rat brain stained for cell bodies **(C)** according to Merker or for myelin **(D)** according to Gallyas depict the needle tract (arrow) and the needle tip (asterisk) of a BoNT-A-injection ipsilateral to the 6-OHDA-application (9 months survival). Scale bar applies to **(C,D)**: 2 mm.

Surgery was conducted under aseptic conditions under ketamine (50 mg/kg body weight)/xylazine (4 mg/kg body weight) anesthesia using a stereotactic frame (David Kopf Instruments). For nearly complete lesion of the right side substantia nigra compact part 4 μl 6-OHDA solution (24 μg, Sigma-Aldrich, St. Louis, MO, USA) dissolved in 0.1 M citrate buffer was injected over 4 min via a 26 gauge 5 μl Hamilton syringe into the MFB. The injection coordinates with reference to bregma were: anterior-posterior = −2.3 mm, lateral = 1.5 mm and ventral = −9.0 mm, respectively (Paxinos and Watson, 2007; Figures 1A,B). The success of the lesion was evaluated with apomorphine-induced rotations 1 month after surgery. All animals displayed more than four contralateral rotations/min, indicating a unilateral death of about 97% of the nigrostriatal DAergic neurons (Ungerstedt and Arbuthnott, 1970) and, therefore, were tested further. As reported previously (Wree et al., 2011; Hawlitschka et al., 2013), 6 weeks after 6-OHDA-lesioning animals received injections of 2 × 1 μl BoNT-A solution (lot No. 13028A1A; List, Campbell, CA; purchased via Quadratech, Surrey, UK) containing a total of 1 ng BoNT-A dissolved in phosphate-buffered saline with 0.1% bovine serum albumin (PSA-BSA 0.1%) added into the right (ipsilateral) or left (contralateral) CPu. The coordinates with reference to bregma for the ipsilateral BoNT-A-application were: anterior = +1.3/−0.4 mm, lateral 2.6/3.6 mm to the right, and ventral −5.5 mm, respectively, those for the contralateral BoNT-A-application were: anterior = +1.3/−0.4 mm, lateral 2.6/3.6 mm to the left, and ventral −5.5 mm (Figures 1A,B). Coronal sections stained for cell bodies (Merker, 1983) and myelin (Gallyas, 1971, 1979) depict injection site at −0.4 mm, 3.6 mm and −5.5 mm (Figures 1C,D).

### Behavioral Testing

#### Drug-Induced Rotation Tests (Apomorphine, Amphetamine)

**Figure 2:**
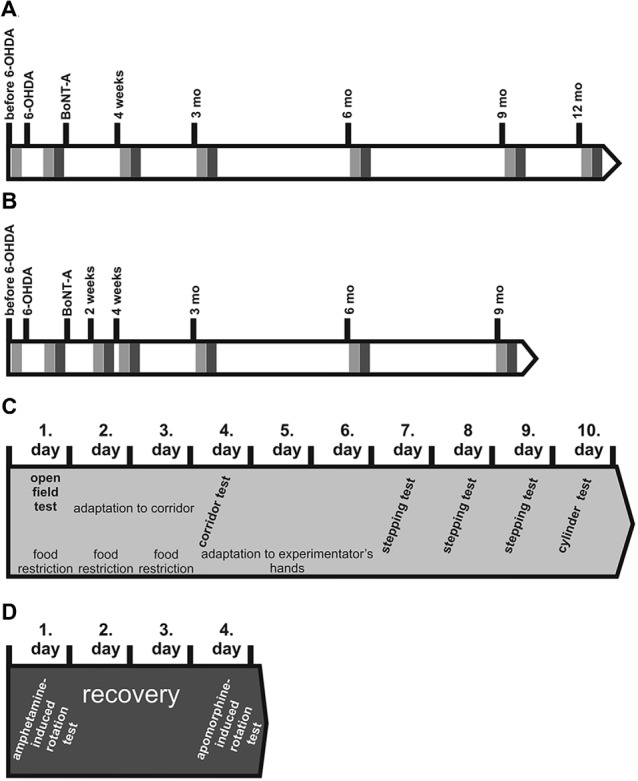
Time points of lesions and behavioral tests in rats BoNT-A or sham-injected **(A)** ipsilateral or **(B)** contralateral to 6-OHDA. Light gray rectangles symbolize batteries of spontaneous behavior tests performed subsequently: open field (OF) test, corridor task, stepping and cylinder tests. Dark gray rectangles symbolize amphetamine-induced rotation test followed by an apomorphine-induced rotation test 3 days later. **(C)** The battery of spontaneous behavior tests were performed as follows: each behavior test series lasted 10 days. At the first day rats were tested for 10 min in the OF arena. Thereafter, rats were food restricted and adapted 2 days rats to the corridor task apparatus for 10 min each. One day later the final corridor test was carried out for 5 min. At the following 3 days rats were handled by the experimentator for 5 min each day. On the following 3 days rats underwent the stepping test twice a day. On the 10th day the forepaw usage was evaluated by the cylinder test. **(D)** Finally, drug-induced rotation tests were performed. First amphetamine rotation test was done for 60 min. Three days later rats were tested for apomorphine-induced rotations for 40 min.

**Figure 3:**
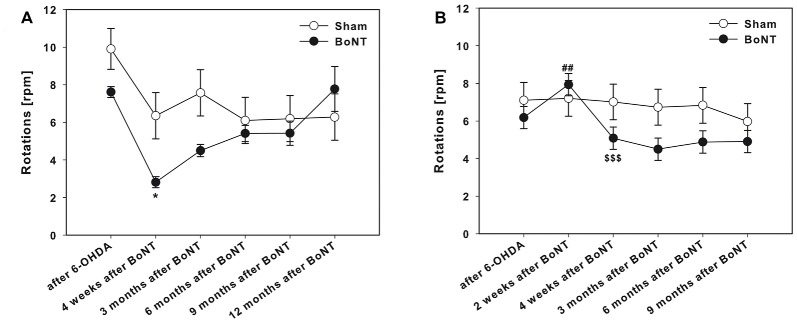
Apomorphine-induced rotations in hemi-Parkinson’s disease (PD) rats treated with intrastriatal BoNT-A- or vehicle-applied **(A)** ipsilaterally or **(B)** contralaterally. Ipsilateral BoNT-A-injections significantly decreased anti-clockwise rotations 4 weeks and 3 months compared to sham injection. Contralateral BoNT-A-injection caused a significant increase of the turning rate only 2 weeks after surgery. Asterisks indicate significant differences compared to the sham group (*P < 0.05). Hashtags indicate significant differences compared to values before BoNT-A-injection (^##^*P* < 0.01). Paragraphs indicate significant differences compared to the previous value after BoNT-A-injection (^§§§^*P* < 0.001). Data are represented as mean ± SEM.

**Figure 4:**
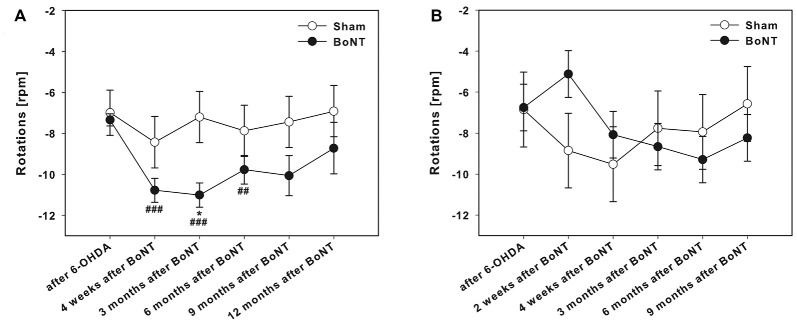
Amphetamine-induced rotations in hemi-PD rats treated with intrastriatal BoNT-A-or vehicle-applied **(A)** ipsilaterally or **(B)** contralaterally. Ipsilateral BoNT-A-injection caused significantly increased rotations 4 weeks to 6 months after BoNT-A, while sham-injected rats did not change rotational behavior. Contralateral BoNT-A-injection in hemi-PD rats caused no significant change of rotational behavior compared to the sham group. Asterisks indicate significant differences compared to the sham group (*P < 0.05). Hashtags indicate significant differences compared to values before BoNT-A-injection (^##^*P* < 0.01, ^###^*P* < 0.001). Data are represented as mean ± SEM.

Rotations were assessed using an automated, self-constructed rotometer system based on the design of Ungerstedt and Arbuthnott (1970) and defined as complete 360° turns and registered as net differences between the two directions per minute. Rotational behavior was induced by amphetamine and apomorphine in hemi-PD rats injected with BoNT-A. Tests were performed prior to (i.e., 4 weeks after 6-OHDA-lesion) and at five time points (2 weeks and 1, 3, 6 and 9 months) after intrastriatal application of BoNT-A (Figure 2). Animals were injected with d-amphetamine sulfate (2.5 mg/kg, s.c., Sigma Aldrich) and monitored for 60 min and in each case 3 days later with apomorphine (0.25 mg/kg, i.p.; Teclapharm, Germany), followed by registration of rotation for 40 min. In the hemi-PD rats, apomorphine-induced anti-clockwise rotations were expressed by positive values (Figure 3), whereas amphetamine-induced rotations in clockwise direction were expressed by negative values (Figure 4).

#### Spontaneous Motor Tests

Corridor task, stepping and open field (OF) tests were realized before 6-OHDA-lesion and 4 weeks thereafter, and 4 weeks, 3 and 6 months after ipsilateral intrastriatal injection of BoNT-A or vehicle, or 2 and 4 weeks, 3, 6 and 9 months after contralateral intrastriatal injection of BoNT-A or vehicle (Figure 2). Cylinder test was performed after 6-OHDA-lesion and 4 weeks, 3, 6, 9 and 12 months after ipsilateral intrastriatal injection of BoNT-A or vehicle, or before 6-OHDA-lesion and 4 weeks thereafter, and 2 and 4 weeks, 3, 6 and 9 months after contralateral intrastriatal injection of BoNT-A or vehicle (Figure 2).

#### Stepping Test

Forelimb akinesia was assessed using a modified version of a stepping test (Olsson et al., 1995) primary described as bracing test by Schallert et al. (1979) and has been established as a sensitive measure of bradykinesia in unilateral 6-OHDA-lesioned rats (Schallert et al., 1992; Lindner et al., 1997). We evaluated the adjusting steps. Rats were handled by the experimenter during 3 days to become familiar with the test procedure. Thereafter, tests were performed twice per day on three consecutive days. Briefly, the rat was held by the investigator with one hand softly blocking both its hind limbs and the not monitored forelimb, the unrestrained forepaw touching the table. In doing so the rat was moved slowly sideways across the table (0.9 m in 5 s) and the number of adjusting steps of the respective unrestrained left or right forepaw was counted while moving in the forehand and backhand directions. Finally, the means of forehand and backhand steps of left and right paws were calculated.

#### Cylinder Test

Forelimb preference was evaluated with the cylinder test in both groups. The use of the left and right forepaws during vertical exploration in a glass cylinder with a diameter of 20 cm was documented and analyzed with a video camera system (Sony) according to Schallert and Tillerson (2000) and Kirik et al. (2001). Thirty consecutive forepaw contacts with the glass cylinder were evaluated per animal by counting the initial contacts of the right or left paw and calculating the ratio of left and right forepaw use. To prevent subjective bias, contacts made by each forepaw with the cylinder wall were scored from videotapes by an observer blinded to the animals’ identities.

#### Open Field Test

Spontaneous horizontal locomotor activity and anxiety were estimated via the OF test (Hall and Ballachey, 1932; Hall, 1934). Rats were placed in a square OF arena of 50 × 50 cm, which was positioned inside an isolation box (TSE-Systems, Bad Homburg, Germany). Rats were adapted for 1 h before the test at dark-phase in the examination room. Illumination of the OF test was provided by a white photo bulb at 450 lx and animals were monitored online by a video camera placed inside the isolation box and tracked using the VideoMot2 Software (TSE Systems). The OF was divided into 16 quadratic subfields in 12 peripheral and four central area by a grid in the tracking software. This paradigm mimicked the natural conflict in rats between the tendency to explore a novel environment and the tendency to avoid a brightly lit open arena (DeFries et al., 1966; Eikelis and Van Den Buuse, 2000). The rats were tested once in the arena for 10 min. Environmental odors were removed by cleaning the OF after each session to avoid influences of the behavior by odor trials. The total running distance of the animals, time spend in the center and in the edges of OF arena (Andringa et al., 2000), also the ration of center distance to total distance (Denenberg, 1969) were evaluated.

#### Corridor Task

Lateralized sensorimotor integration and neglect for the side contralateral to the 6-OHDA-lesion were examined using the adjacent version of corridor task according to Grealish et al. (2010). Before testing rats went onto a food restriction diet for 3 days and maintained at 90% of free-feeding bodyweight during habituation and testing (Schackel et al., 2013). Animals were adapted into the apparatus, a long, narrow self-constructed alleyway (240 cm long × 7 cm wide × 23 cm deep) for 10 min each on two consecutive days with some scattered sugar pellets (Ain-76A Rodent Tablet 20 mg TestDiet, Richmond, IN, USA) along the floor of the corridor and started from different ends of the corridor each day. On the test day, rats were first positioned individually in an identical, but empty corridor for 5 min for adaptation and then placed to the end of the testing corridor in which bowls (2 cm in diameter, distance between the bowls 15 cm) containing 5 pellets placed on the left and right sides. Rats were free to retrieve pellets from either side of their body for 5 min (Fitzsimmons et al., 2006). The number of ipsilateral (right side) and contralateral (left side) retrievals was counted and the data were expressed as the percentage of left or right side retrievals on the total number of retrievals. A “retrieval” involved a nose poke into a bowl, whether or not any pellets were taken from it, defining the side according to the rat’s body axis (Dowd et al., 2005; Döbrössy and Dunnett, 2007; Grealish et al., 2010).

#### Statistics

Data of behavioral tests was subjected to two-way ANOVA with repeated measurements. A one-way repeated measures ANOVA is used for comparison of different time points in separate treatment groups which is essentially the same design as a paired *t*-test. The Holm-Sidak approach was used for adjustment for multiple testing for *post hoc* comparisons. A critical value for significance of *P* ≤ 0.05 was used throughout the study. In case of non-normally distributed data, data were subjected to Kruskal-Wallis one- or two-way ANOVA on ranks. Dunn’s test was used for *post hoc* comparisons after ANOVA on ranks to adjust for multiple testing. All statistical tests were done using SigmaPlot 11 Software (Supplementary Table S1).

## Results

### Apomorphine-Induced Rotations

The right side hemi-PD rats showed apomorphine-induced anti-clockwise rotations of about 6–10 rotations per minute (Figures 3A,B).

#### Ipsilateral BoNT-A-Injection

Ipsilateral BoNT-A-injection caused a significant decrease in rotational behavior 4 weeks and 3 months after BoNT-A, thereafter rotations of BoNT-A-injected rats equaled those after sham-injection (Figure 3A).

#### Contralateral BoNT-A-Injection

The injection of 1 ng BoNT-A into the left striatum of right sided 6-OHDA-lesioned rats caused a significant increase of the turning rate 2 weeks after surgery. Remarkably, already 2 weeks later, i.e., 1 month after BoNT-A-administration, the turning rate decreased to a level, which was not significantly different from the turning rate prior to BoNT-A treatment (Figure 3B).

### Amphetamine-Induced Rotations

The hemi-PD rats showed amphetamine-induced clockwise rotations of about seven turns per minute (Figures 4A,B).

#### Ipsilateral BoNT-A-Injection

Ipsilateral intrastriatal BoNT-A-injection caused a significantly increase of the turning rate 4 weeks to 6 months after BoNT-A (Figure 4A). Sham-injected rats did not change rotational behavior in hemi-PD rats (Figure 4A).

#### Contralateral BoNT-A-Injection

Contralateral intrastriatal BoNT-A-injection in hemi-PD rats caused a tentative decrease of rotational behavior only at 2 weeks after BoNT-A (Figure 4B). At all other post-BoNT-A-injection time-points BoNT-A- and sham-injected rats did not differ in rotational behavior in hemi-PD rats (Figure 4B).

### Stepping Test

**Figure 5:**
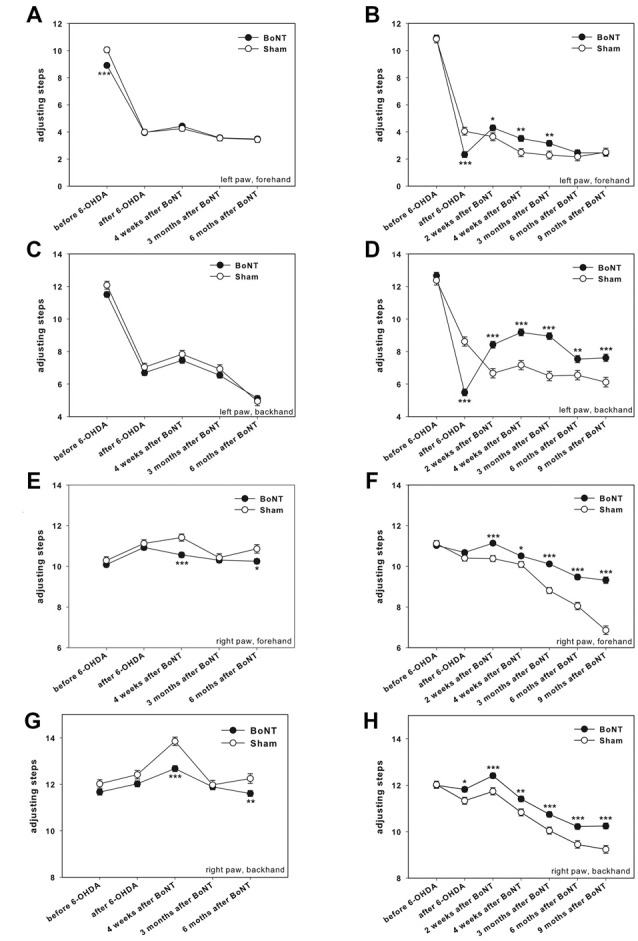
Stepping test in right side hemi-PD rats treated with intrastriatal BoNT-A- or vehicle-applied **(A,C,E,G)** ipsilaterally or **(B,D,F,H)** contralaterally. In unlesioned rats 9–12 adjusting steps for the left **(A–D)** and right **(E–H)** forelimbs in forehand and backhand directions were seen. In hemi-PD rats the use of the left forepaw was impaired in both the forehand **(A,B)** and backhand **(C,D)** directions. Neither ipsilateral BoNT-A nor sham injection changed the impairments of left paw steps in hemi-PD rats **(A,C)**. The stepping of the right forelimb in hemi-PD rats in both the forehand **(E)** and backhand **(G)** directions were generally not affected by ipsilateral BoNT-A or sham injection. Contralateral BoNT-A-injected hemi-PD rats significantly improved left paw forehand steps from 2 weeks to 3 months after BoNT-A **(B)**, and backhand steps from 2 weeks until 9 months after BoNT-A **(D)**. Right forepaw adjusting steps significantly increased from 2 weeks up to 9 months after BoNT-A-application as well in forehand **(F)** as in backhand **(H)** directions compared to sham-injected rats. Asterisks indicate significant differences compared to the sham group (*P < 0.05, **P < 0.01, ***P < 0.001). Data are represented as mean ± SEM.

Adjusting steps were measured on the unlesioned and lesioned sides for each group in the forward and backward directions. Before 6-OHDA-lesion no difference in the number of adjusting steps for the left and right forepaws in forehand and backhand directions for both experimental groups were observed (Figures 5A–H). Rats of both groups with their left and right forepaws made about 9–12 steps both in forward and backward directions. After 6-OHDA-lesion only the performance of the left forepaw (contralateral to 6-OHDA) was impaired in both the forehand (Figures 5A,B) and backhand (Figures 5C,D) directions.

#### Ipsilateral BoNT-A-Injection

Neither ipsilateral BoNT-A nor sham injections changed the impairments of hemi-PD rats. At the same time, side stepping movements of the right forepaw in 6-OHDA-lesioned rats in both the forehand and backhand directions were generally neither affected by ipsilateral BoNT-A nor sham injection up to 6 months (Figures 5E,G), with exception of single time points.

#### Contralateral BoNT-A-Injection

As compared to sham-injected rats, the BoNT-A-injected animals significantly improved left paw forehand steps from 2 weeks to 3 months after BoNT-A (Figure 5B), and backhand steps from 2 weeks until 9 months after BoNT-A (Figure 5D). In addition, we found significantly more adjusting steps of the right forepaw from 2 weeks up to 9 months after BoNT-A-application as compared to sham-injected rats, as well in forehand (Figure 5F) as in backhand (Figure 5H) directions. Thus, contralateral BoNT-A-injection positively influences forelimb usage in hemi-PD rats. Moreover, there was an age-related decrease in right paw stepping (Figures 5F,H).

### Cylinder Test

**Figure 6:**
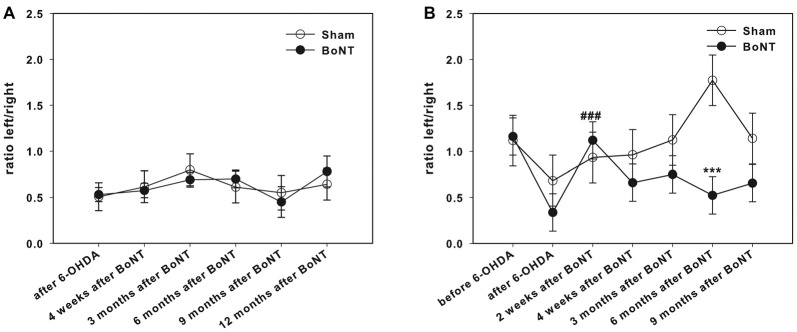
Cylinder test in right side hemi-PD rats treated with intrastriatal BoNT-A- or vehicle-applied **(A)** ipsilaterally or **(B)** contralaterally. Hemi-PD rats exhibited a significantly reduced use of the left forelimb resulting in left/right ratio of about 0.5 **(A,B)**. Neither ipsilateral BoNT-A nor sham injection showed any significant improvement. Contralateral intrastriatal injection of BoNT-A only led at 2 weeks after BoNT-A to a significant readjustment of the right and left forepaw usage. Hashtags indicate significant differences compared to values before BoNT-A-injection (^###^*P* < 0.001). Asterisks indicate significant differences compared to the sham group (***P < 0.001). Data are represented as mean ± SEM.

Right side hemi-PD rats exhibited a significantly reduced use of the left forelimb, resulting in an about 50% decrease of the left/right ratio of forelimb usage (Figures 6A,B).

#### Ipsilateral BoNT-A-Injection

Intrastriatal ipsilateral treatment of hemi-PD rats with 1 ng of BoNT-A did not show any significant improvement of the left forelimb usage as assessed by the cylinder test. Also sham-treated rats showed no significant effect in forelimb usage of hemi-PD rats (Figure 6A).

#### Contralateral Injection

Interestingly, intrastriatal injection of BoNT-A contralateral to the lesioned side led 2 weeks after BoNT-A-application to a significant readjustment of the left and right forepaw usage. In the course of the following months this effect decreased (Figure 6B).

### Open Field

**Figure 7:**
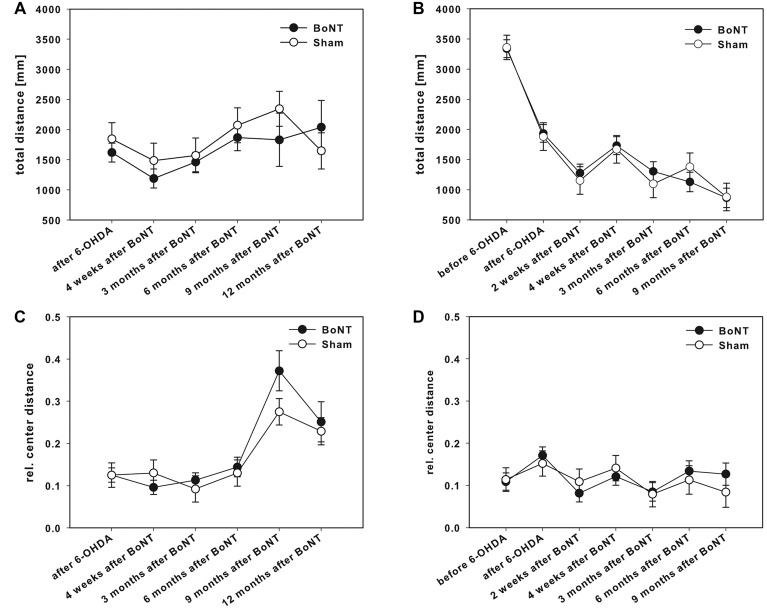
OF test in right side hemi-PD rats treated with intrastriatal BoNT-A- or vehicle-applied **(A,C)** ipsilaterally or **(B,D)** contralaterally. Rats of both BoNT-A- and sham-injected groups showed decreased locomotor activity compared to prelesion results **(B)**. Neither ipsilateral BoNT-A nor sham injection showed any significant changes in total running distance **(A)** or the ratio of center distance to total distance **(C)**. The same is true for both contralaterally injected groups **(B,D)**. Data are represented as mean ± SEM.

Hemi-PD rats of both BoNT-A- and sham-injected groups showed decreased locomotor activity compared to prelesion results (Figure 7B).

#### Ipsilateral BoNT-A-Injection

The total running distance (Figure 7A) and also the ratio of center distance to total distance (Figure 7C) were not significantly different between the BoNT-A and the sham group over time. Moreover, ipsilateral BoNT-A-injected rats and sham group spent the same time in the edges of the OF apparatus (data not shown).

#### Contralateral BoNT-A-Injection

The total running distance was similar in both experimental groups after contralateral application of BoNT-A or sham treatment up to 9 months (Figure 7B). Also, the relative center distance (Figure 7D) of the BoNT-A-injected animals did not differ significantly from the sham group over time as did the times spent in the edges of the OF (data not shown).

### Corridor Task

**Figure 8:**
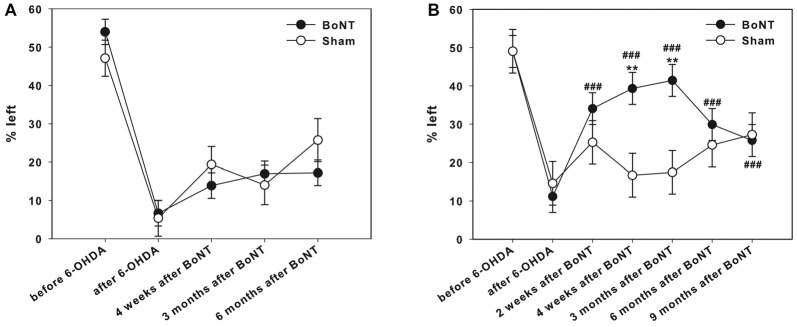
Corridor task in hemi-PD rats treated with intrastriatal BoNT-A- or vehicle-applied **(A)** ipsilaterally or **(B)** contralaterally. Preoperative screening showed that all animals equally retrieved pellets from either left or right sides **(A,B)**. Right side hemi-PD rats significantly neglected of the left corridor side. Ipsilateral BoNT-A as well as sham injections did not improve contralateral sensorimotor integration in hemi-PD rats. Contralateral BoNT-A-injection reversed this bias in hemi-PD rats 2 weeks to 9 months. Significant contralateral retrievals of about 40% were seen. Left side sham-injected rats did not improve in this task over time. Asterisks indicate significant differences compared to the sham group (**P < 0.01). Hashtags indicate significant differences compared to values before BoNT-A-injection (^###^*P* < 0.001). Data are represented as mean ± SEM.

Two weeks before lesion surgery, rats were tested in the adjacent version of the corridor task. This preoperative screening showed that all animals equally retrieved pellets from either left or right sides (Figures 8A,B). The right side 6-OHDA-lesion caused a significant neglect of the left corridor side; only about 5% of left retrievals were measured (Figures 8A,B).

#### Ipsilateral BoNT-A-Injection

Ipsilateral intrastriatal BoNT-A as well as sham injections in hemi-PD rats did not improve contralateral sensorimotor integration up to 6 months significantly (Figure 8A).

#### Contralateral BoNT-A-Injection

Left side sham-injected rats did not improve in this task over time. In contrast, contralateral intrastriatal BoNT-A-injection significantly reversed this bias 2 weeks to 9 months after treatment. Significant contralateral retrievals of about 40% were seen. However, 6 and 9 months after BoNT-A values declined again and equaled those of sham-injected rats (Figure 8B).

## Discussion

In our previous publications (Wree et al., 2011; Holzmann et al., 2012; Antipova et al., 2013; Hawlitschka et al., 2013; Mehlan et al., 2016), we described the effect of intrastriatal injection of BoNT-A in a rat model of hemi-PD. We used the 6-OHDA to induce hemi-PD, a widely utilized and highly reproducible toxin-based animal model of PD in rats (Ungerstedt et al., 1974; Schwarting and Huston, 1996; Deumens et al., 2002; Blandini and Armentero, 2012). Unilateral 6-OHDA-injection of the right MFB that conveys the efferent fibers from nigral cell bodies to the striatum, causes massive degeneration of the nigrostriatal pathway, highest level of nigral cell loss and striatal DA depletion (over 90%; Dauer and Przedborski, 2003; Blandini and Armentero, 2012). The resulting motor deficits at the side of the body contralateral to the lesion (Deumens et al., 2002) can be evaluated by spontaneous and drug-induced behavioral phenotypes. As described originally by Ungerstedt and Arbuthnott (1970), a 6-OHDA-lesion of the right SNC causes anti-clockwise, i.e., contraversive apomorphine-induced rotations (Ungerstedt et al., 1969). The most important finding in our prior studies with intrastriatally applied BoNT-A was the complete abolition of apomorphine-induced rotations up to 6 months, when the rats received 1 ng of BoNT-A into the right striatum 6 weeks after right side 6-OHDA-lesion (Wree et al., 2011; Antipova et al., 2013).

In continuation of previous studies and to enhance our knowledge of the BoNT-A-injection model, we here examined the effects of BoNT-A-injection into the striatum as well ipsilateral as contralateral to the hemisphere that received 6-OHDA before. For that reason we used a broad spectrum of different behavior tests, including spontaneous test, i.e., corridor task, stepping, cylinder and OF test and drug-induced motor tests, i.e., apomorphine- and amphetamine-induced rotations, to characterize and compare the effects of ipsilateral and contralateral intrastriatal BoNT-A-injections on the motor deficits, lateralized neglect, bradykinesia, emotionality and anxiety in hemi-PD rats.

We found a differentiated behavioral profile after ipsilateral or contralateral BoNT-A-application in hemi-PD rats. The effects of ipsilaterally injected BoNT-A were in line with those published previously (Wree et al., 2011; Holzmann et al., 2012; Antipova et al., 2013; Hawlitschka et al., 2013; Mehlan et al., 2016). Interestingly, the effects of contralaterally injected BoNT-A were either changed to the opposite compared to ipsilateral BoNT-A, or gave results not known yet. The disruption of presynaptic ACh as well as DA release by BoNT-A is transient and the synaptic signal transmission was shown by forming of new intact SNARE complexes within several months (Sweeney et al., 1989; Shapovalova, 1995; Washbourne et al., 1998; Weinstock et al., 2002). In the following paragraphs, the results seen in the various behavioral parameters are discussed with respect to assumed or hypothetical changes of transmitter and receptor concentrations in the CPu as a key structure of the basal ganglia loops induced by dopaminergic deprivation and BoNT-A-application.

### Basal Ganglia Circuitry in Hemi-PD Rats

Dopaminergic deprivation of the striatum is followed by reactive adaptations of transmitter receptors, mainly by an upregulation of D2 receptors in the range of about 20%–30% (Reader and Dewar, 1999; Choi et al., 2012; Sun et al., 2013; Konieczny et al., 2017). The majority of inhibitory D2 receptors are located on cholinergic interneurons and medium spiny neurons (MSN) projecting to the external pallidum, i.e., being part of the indirect basal ganglia loop (Hurley and Jenner, 2006; Perreault et al., 2011; Bordia et al., 2016; Mamaligas et al., 2016; Rico et al., 2017). As in hemi-PD the tonically active cholinergic interneurons become hyperactive due to loss of D2-mediated DA inhibition, they strongly activate MSN by ACh. Thus, complementary to the loss of inhibitory D2-mediated DA inhibition the MSN increase their firing intensity also by cholinergic hyperstimulation. The increased GABAergic MSN projection reaches the external globus pallidus (EGP). As the EGP is thus strongly inhibited, its GABAergic projection to the internal globus pallidus (IGP = entopeduncular nucleus) and mainly to the subthalamic nucleus (STh) is strongly reduced compared to normal. As a result, the spontaneously active STh is less inhibited and thus more intensely firing, further stimulated by normal cortical glutamatergic afferents. As a consequence, the STh using glutamate as transmitter can stimulate the IGP more intensely. The massively stimulated IGP projects using GABA as transmitter to the ventrolateral thalamic nucleus (VL) that reacts with an inhibition of its neurons. Finally, the inhibited VL neurons send less activating glutamatergic stimuli to the premotor cortex that in turn reacts with a reduced initiation of movements of the contralateral body via its crossed motor efferents (Obeso et al., 2008a,b, 2014). These changes in basal ganglia circuitry seem to underlie the movement initiation deficits, the akinesia and the reduced spontaneous use of the contralateral forelimb and changed walking pattern in hemi-PD rats (Blandini et al., 2000; DeLong and Wichmann, 2007; Braak and Del Tredici, 2008; Quiroga-Varela et al., 2013; Tremblay et al., 2015).

### Drug-Induced Behavior

#### Apomorphine-Induced Rotations

Unilateral dopaminergic depletion led to apomorphine-induced contralateral rotations, ipsilateral intrastriatal BoNT-A reduced rotational behavior for at least 6 months, and contralateral intrastriatal BoNT-A increased rotational behavior only shortly at 2 weeks after BoNT-A in hemi-PD rats.

In hemi-PD rats apomorphine-induced rotations are due to binding of apomorphine to the upregulated D2 receptors in the dopaminergically deprived CPu neurons (Konieczny et al., 2017). Inhibitory D2 receptors are mostly located on cholinergic interneurons and MSN projecting to the EGP, i.e., being part of the indirect basal ganglia loop (Pisani et al., 2007; Tozzi et al., 2011; Lim et al., 2014; Bordia et al., 2016; Mamaligas et al., 2016). In hemi-PD rats apomorphine deactivates the hyperactive cholinergic interneurons via D2 receptors and also inhibits the D2-bearing MSN, forming the central parts of the indirect basal ganglia loop and projecting with reduced intensity to the EGP. As the EGP is disinhibited, its GABAergic projection to the STh is increased compared to normal and thus the more inhibited STh can stimulate the IGP less intensively. The less active IGP projects less intensively to the VL, and consequently, the disinhibited VL sends much more activating stimuli to the premotor cortex, which in turn reacts with an increased initiation of movements and muscle innervation of the contralateral body side. Thus, apomorphine induces contralateral rotations in hemi-PD rats.

Ipsilateral intrastriatal application of 1 ng BoNT-A in hemi-PD rats completely abolished apomorphine-induced contralateral rotation behavior for at least 6 months. BoNT-A is thought to block the ACh release of cholinergic interneurons, which are hyperactive in PD (Day et al., 2006; Obeso et al., 2008a,b). Seemingly, ipsilateral BoNT-A normalized ACh concentration in the injected CPu for a limited time, i.e., up to 6 months until BoNT-A is metabolized. Moreover, jet unpublished own results of BoNT-A-induced receptor concentration measurements speak in favor of a BoNT-A-induced reduction of D2 receptors in the respective striatum. Following the indirect basal ganglia loop the reduced activity of the D2 receptor bearing MSN result in a downregulation, i.e., normalization of the VL activity and the abolition of apomorphine-induced contralateral rotation behavior. These effects seemed to exist for at least 3 months, thereafter BoNT should be metabolized.

Contralateral intrastriatal application of BoNT-A shortly increased apomorphine-induced contralateral rotation behavior only at 2 weeks. It can be hypothesized that BoNT-A shortly reduced the D2 receptors in the contralateral striatum thus increasing the difference in the D2 receptor concentrations between both hemispheres and by this leads to an increase of the number of rotations.

#### Amphetamine-Induced Rotations

Unilateral dopaminergic depletion led to amphetamine-induced ipsilateral rotations, ipsilateral intrastriatal BoNT-A increased rotational behavior for at least 6 months, and contralateral intrastriatal BoNT-A tentatively decreased rotational behavior shortly at 2 weeks after BoNT-A in hemi-PD rats.

Amphetamine as a DA releaser causes strong DA delivery in the CPu contralateral to the 6-OHDA-lesion and a low one in the ipsilateral striatum because of the loss of DA afferents. Consequently, the strong DA imbalance activates the contralateral inhibitory dopaminergic system, influencing the respective MSN via ACh, and by this causes ipsilateral rotations (about 7 per min). Moreover, the massive innervation of the D1 receptor bearing MSN in the CPu contralateral to the DA depletion also results in a stimulation of that hemisphere resulting in clockwise rotations of right side hemi-PD rats.

Ipsilateral intrastriatal application of 1 ng BoNT-A in hemi-PD rats increased amphetamine-induced ipsilateral rotation behavior for at least 6 months. Apparently, BoNT-A blocked the ACh release of cholinergic interneurons and reduced D2 receptor concentration in the respective striatum. Moreover, intrastriatal application of BoNT-A could have altered the concentration of other transmitters and their receptors, too (Bigalke et al., 1985; Ashton and Dolly, 1988; Poulain et al., 1990). As it is described that amphetamine and its derivates are able to bind directly to α-2 adrenergic receptors (Ritz and Kuhar, 1989) and activate serotonin receptors (Nichols et al., 1994; Schmidt et al., 1994), these receptors could also play a role in the BoNT-A-induced changes in amphetamine-induced rotation behavior.

Amphetamine-induced ipsilateral rotations were tentatively and shortly decreased at 2 weeks after contralateral intrastriatal BoNT-A-application. Possibly, BoNT-A temporally reduced DA release in the injected contralateral striatum thus decreasing the difference in the DA concentrations between both hemispheres and by this leading to a decrease of the number of rotations.

### Spontaneous Motor Tests

#### Stepping Test

Unilateral right side dopaminergic depletion profoundly impaired stepping performance of the left paw (contralateral to 6-OHDA-lesion) in both forehand and backhand directions. Ipsilateral intrastriatal BoNT-A did not change the impairments seen in hemi-PD rats, while contralateral intrastriatal BoNT-A improved left paw forehand and backhand steps and positively influenced right forelimb usage.

In hemi-PD rats our results confirmed those of many others (Schallert et al., 1992; Mukhida et al., 2001; Kelsey et al., 2004; Tseng et al., 2005; Manfredsson et al., 2007; Pioli et al., 2008; Fang et al., 2010; Seeger-Armbruster and von Ameln-Mayerhofer, 2013; Shin et al., 2014; Tronci et al., 2015) showing the severe impairment of the contralateral paw in combination with unaltered stepping performance of the ipsilateral paw (Winkler et al., 1996; Kelsey et al., 2004; Pinna et al., 2010). The motor initiation deficits in the forelimbs, analogous to limb akinesia and gait problems in PD patients, were produced by DA depletion (Sabol et al., 1985; Fairley and Marshall, 1986; Salamone et al., 1993). As dopaminergic deprivation of the striatum is followed by increased GABAergic MSN projection to the EGP, resulting in a disinhibition of the spontaneously active STh and, as a result a more actively firing IGP, which inhibits VL. Finally, the inhibited VL neurons do not sufficiently activate the premotor cortex that in turn reduces initiation of movements of the contralateral body via its crossed motor efferents (Obeso et al., 2008a,b, 2014; Wree and Schmitt, 2015).

Ipsilateral intrastriatal application of 1 ng BoNT-A in hemi-PD rats did not change the impairments seen in hemi-PD rats. Obviously, the BoNT-A-induced changes in the DA-deprived CPu concerning extracellular ACh content, and DA and other transmitter receptors are not sufficient to improve spontaneous motor tasks. Importantly, several reports emphasized that it is the striatal DA depletion of over 80% that is responsible for decreased movement initiation (Sabol et al., 1985; Fairley and Marshall, 1986; Salamone et al., 1993; Chang et al., 1999; Seeger-Armbruster and von Ameln-Mayerhofer, 2013; Sun et al., 2013). As in our hemi-PD rats the DA depletion was nearly complete (Barnéoud et al., 2000; Kelsey et al., 2004), the ipsilateral intrastriatal BoNT-A-application is without effect on stepping tasks.

Contralateral intrastriatal application of 1 ng BoNT-A in hemi-PD rats improved the impairments of the left paw seen in hemi-PD rats for 3 (forehand steps) or 9 (backhand steps) months. This phenomenon is not fully understood. D1 and D2 receptor binding contralateral to unilateral 6-OHDA-lesion is reported unchanged to normal rats (Lawler et al., 1995; Pelled et al., 2002), whereas D2 receptor concentration is generally increased ipsilaterally (Araki et al., 1998; Reader and Dewar, 1999; Choi et al., 2012; Sun et al., 2013; Konieczny et al., 2017). This is corroborated by Capper-Loup et al. (2013) stating that contralateral to the 6-OHDA-lesion striatal D1R mRNA and D2R mRNA resemble those of naive rats, whereas ipsilaterally D1R mRNA is reduced and D2R mRNA is increased (Capper-Loup et al., 2013). With respect to DA concentration in the contralateral hemisphere contradicting measures were reported. Fox et al. (2016) found striatal DA release from the contralateral unlesioned substantia nigra equivalent in lesioned and control rats, whereas Zetterström et al. (1986) dialyzed more DA contralaterally to 6-OHDA than in unleased controls (Zetterström et al., 1986; Fox et al., 2016). Several groups hypothesized that if it is likely that the unilateral lesion of the nigrostriatal pathways affects both sides of the brain (Lawler et al., 1995; Fox et al., 2016), then it is not yet clear how the unlesioned side is influenced. It is discussed that Interhemispheric projections have functional significance (Fox et al., 2016; Schmitt et al., 2016). Lawler et al. (1995) combined a 6-OHDA with a corpus callosum transection in order to minimize interhemispheric projections on possible changes in the D1 and D2 receptors, DA and its metabolites in the non-lesioned striatum (Lawler et al., 1995). Bilateral cortico-striatal projections were traced in rats (Hassler et al., 1982; Berendse et al., 1992), however with ipsilateral predominance: both cortices project to both striata (Hassler et al., 1982; Berendse et al., 1992; Lieu and Subramanian, 2012; Schmitt et al., 2016), i.e., intact cortex to the lesioned striatum and cortex of the lesioned hemisphere to the intact striatum (Lieu and Subramanian, 2012) arguing that interhemispheric connections can influence behavioral responses to nigrostriatal lesioning. Unfortunately, there is no comprehensive summary of changes in neuroanatomical and electrophysiological properties in the so-called healthy hemisphere induced by unilateral lesion of the nigrostriatal dopaminergic system and its correlation to behavior.

Contralateral intrastriatal application of 1 ng BoNT-A in hemi-PD rats improved the use of the right paw in hemi-PD rats for up to 9 months both in the number of forehand and backhand steps. In this case, BoNT-A was injected into a seemingly normal transmitter and receptor environment. BoNT-A is thought to block the ACh release of cholinergic interneurons and DA release of dopaminergic afferents (Bigalke et al., 1985; Ashton and Dolly, 1988), perhaps also reduces the D2 receptor concentration. As a result, the MSN decreased the GABAergic innervation of the EGP, resulting in an increased inhibition of the spontaneously active STh and followed by a less actively firing IGP, which inhibits VL to a lesser degree than normally. Finally, the disinhibited VL neurons considerably activated the premotor cortex that in turn increased the initiation of movements of the contralateral body, i.e., the forelimb ipsilateral to the DA deprivation, via its crossed motor efferents. In the end, the paw of the DA deprived body side is hyperactive as compared to the sham-injected hemi-PD rats. As already supposed by Olsson et al. (1995) we observed an age dependent decline of right paw forehand and backhand steps, both in sham- and BoNT-A-injected rats up to 9 months, suggesting some degree of habituation to the test.

#### Cylinder Test

Unilateral right side hemi-PD rats exhibited a significantly reduced use of the left forelimb. Intrastriatal ipsilateral BoNT-A or sham treatment did not show any significant improvement of the left forelimb usage. Interestingly, contralateral intrastriatal injection of BoNT-A led to a short term (at test point 2 weeks after BoNT-A) significant readjustment of forepaw usage.

In the hemi-PD rats our results are in line with others (Schallert and Tillerson, 2000; Kirik et al., 2001; Deumens et al., 2002; Cohen et al., 2003; Vercammen et al., 2006; Rauch et al., 2010; de Araújo et al., 2013), the impaired paw being relatively used by about 40% to 50% compared to the unimpaired. As for the stepping impairment the motor initiation deficit for voluntary movements in contralateral forelimb use is discussed as a result of DA depletion (Whishaw et al., 1986; Lundblad et al., 2002; Shi et al., 2004; Schallert and Woodlee, 2005; Wheeler et al., 2014; Sampaio et al., 2017). As dopaminergic deprivation of the striatum causes increased GABAergic MSN projection to the EGP, and via disinhibition of the spontaneously active STh a more actively firing IGP, the inhibited VL neurons do not sufficiently activate the premotor cortex. Consequently, there is a reduces initiation of movements of the contralateral body via its crossed motor efferents (Obeso et al., 2008a,b, 2014; Wree and Schmitt, 2015).

Ipsilateral intrastriatal application of BoNT-A or sham injection in hemi-PD rats did not change the impairments. Our explanation resembles that of the stepping test. For the execution of the voluntary forelimb movements a normal DA content of the CPu is essential (Salamone et al., 1993; Shi et al., 2004; Manfredsson et al., 2007; Woodlee et al., 2008; Plowman et al., 2011; Fleming et al., 2013; Mabandla et al., 2015). BoNT-A did not improve left forelimb use as it could increase DA concentration.

Contralateral intrastriatal application of 1 ng BoNT-A in hemi-PD rats shortly improved the use of the left paw in hemi-PD rats. Seemingly, BoNT-A for a limited time interacts with ACh release from cholinergic interneurons and DA release from dopaminergic afferents (Bigalke et al., 1985; Ashton and Dolly, 1988).

#### Open Field Test

Spontaneous horizontal locomotor activity and anxiety as measured by the OF test showed decreased locomotor activity in hemi-PD rats compared to prelesion results. Ipsilateral intrastriatal BoNT-A and sham injections neither changed total running distance nor the ratio of center distance to total distance significantly over time. Also contralateral intrastriatal BoNT-A or sham injection had a significant effect on locomotor activity of hemi-PD rats.

Unilateral 6-OHDA-injection decreased the overall spontaneous locomotor behavior as measured by total walking distance as already shown by others (Kirik et al., 2001; Tamás et al., 2005; Brown et al., 2011; Abedi et al., 2013; Capper-Loup et al., 2013; da Rocha et al., 2013; Sun et al., 2013; Machado-Filho et al., 2014; Chao et al., 2015; Kumari et al., 2015; Ximenes et al., 2015; Das et al., 2016; Sgroi et al., 2016). Reduced locomotion is tentatively interpreted as a result of depletion of striatal DA (Fearnley and Lees, 1991; Schwarting and Huston, 1996; Alam and Schmidt, 2002; Ferro et al., 2005; Carvalho et al., 2013), although there is no clear explanation why a unilateral DA depletion has such a massive effect (about 50% reduction) on total walking distance as a measure of whole body bradykinesia of the hemi-PD rats. Additionally, we could not observe that the impairments of hemi-PD rats were age and habituation dependent. It has been previously reported that locomotor activity declined in aging hemi-PD rats (Schulz et al., 2002, 2004; Jezek et al., 2003) or due to habituation (Gentsch et al., 1982; Bureš et al., 1983) in OF.

#### Corridor Task

Naive rats equally retrieved pellets from either left or right sides. The right side 6-OHDA-lesion caused a massive neglect of the left corridor side. Ipsilateral intrastriatal BoNT-A did not improve contralateral sensorimotor integration up to 6 months significantly. In contrast, contralateral intrastriatal BoNT-A-injection significantly reversed this bias, and significant contralateral retrievals of about 40% were seen.

Anatomical, biochemical and behavioral evidence indicate that striatum is heterogeneous with respect to function (Divac et al., 1967; Dunnett and Iversen, 1980; Pisa, 1988) and the intact nigrostriatal innervation is crucial for successful integration of sensory and motor function resulting in a coordinated goal-directed behavior (Turner, 1973; Dunnett and Iversen, 1982; Carli et al., 1985, 1989; Fairley and Marshall, 1986). Thus, the corridor task is a sensitive behavioral test for unilateral 6-OHDA-lesion (Boix et al., 2015) measuring the lateralized sensorimotor proprioception and neglect.

Unilateral striatal DA depletion results in a polymodal “neglect” characterized by a failure to orient to contralateral stimuli (Marshall et al., 1971; Ljungberg and Ungerstedt, 1976; Fairley and Marshall, 1986; Brown et al., 2011) and hemi-PD rats failed to orient to tactile, visual or olfactory stimuli presented on the contralateral side of the body (Dowd et al., 2005). Respective abnormalities have already been reported in PD patients (Berardelli et al., 2001). Brown and Robbins suggested that the “striatal neglect” was not due to a failure to localize the stimuli in contralateral space but, rather, resulted from a deficit in directing responses in contralateral space (Brown and Robbins, 1989). Accordingly, Carli et al. (1985) stated that the neglect is not primarily sensory in nature but is evident only when a contralateral response initiation, in our experiments the retrieval of pellets, is required.

In our study naive animals made equivalent numbers (about 50%) of retrievals from both the left or right corridor sides as shown previously by others (Dowd et al., 2005; Fitzsimmons et al., 2006; Döbrössy and Dunnett, 2007; Kerkerian-Le Goff et al., 2009; Jouve et al., 2010). Hemi-PD rats largely neglect sugar pellets encountered on their contralateral side, demonstrating a pronounced ipsilateral retrieval bias (Torres et al., 2008; Cordeiro et al., 2010; Kaindlstorfer et al., 2012; Naughton et al., 2016). As discussed with stepping and cylinder tests, hemi-PD rats showed a severe impairment of motor initiation of the contralateral body including forelimbs and especially in head orientation. Although the hemi-PD rat seemingly detected the contralateral stimulus (sugar pellets in bowl), it could not react due to the impaired initiation, and, consequently, food pellets were retrieved by the unaltered paw of the opposite side. The motor initiation deficits in the forelimbs were discussed as caused by DA depletion (Sabol et al., 1985; Fairley and Marshall, 1986; Salamone et al., 1993; Dowd et al., 2005; Fitzsimmons et al., 2006) with the known consequences in basal ganglia circuitry.

Intrastriatal ipsilateral BoNT-A-injection did not alter the striatal neglect in hemi-PD rats. This is not surprising, as the DA deficit underlying the neglect was not changed by BoNT-application.

As a key finding in the present study, we obtained a significantly positive effect of intrastriatal contralateral BoNT-A-injection on the contralateral neglect in hemi-PD rats. As discussed with the BoNT-induced changes in the stepping test, we have no comprehensive interpretation of the BoNT-induced behavioral benefit. Taken together, as the molecular mechanism underlying the jet unclear lesion-induced compensatory mechanisms in the hemisphere contralateral to the 6-OHDA-lesion is not further substantiated, we cannot explain the beneficial effect in the contralateral intrastriatal BoNT-application on spontaneous motor task of the hemi-PD rats.

## Conclusion

As hypothesized, some of the behavioral effects seen after ipsilateral BoNT-A-application in hemi-PD rats were reversed or even changed to the opposite by contralateral BoNT-A-injection in hemi-PD rats.

In hemi-PD rats, intrastriatal ipsilateral BoNT-A-injection massively influenced the outcome of drug-induced tests. We found a significant decrease in apomorphine-induced rotations and an increase in amphetamine-induced rotations. Moreover, ipsilateral intrastriatal application of BoNT-A showed no effect on the forelimb usage and akinesia, lateralized sensorimotor integration and spontaneous locomotor activity.

However, contralateral intrastriatal BoNT-A-injection preferentially affected spontaneous motor behavior reducing striatal neglect, and also maintained a significant reduction of the akinesia in stepping test. Furthermore, contralateral intrastriatal application of BoNT-A exhibited a short influence on the apomorphine-induced rotations and led to a transient re-adjustment of right and left forepaw usage in cylinder test. Amphetamine-induced rotations and locomotor activity in OF test stayed unaltered after contralateral BoNT-A-injection.

These temporally restricted effects of BoNT-A-application suggest that intrastriatally applied BoNT-A acted not only as an inhibitor of ACh release but also had impact on transmitter receptors, especially D2 receptor expression and thereby on the basal ganglia circuitry. Evaluation of receptor concentrations of all important striatal transmitters are subject of ongoing studies of our group.

## Author Contributions

CH, OS and AW designed the study. VAA, AW and AH performed experiments. CH did the statistics and the figures. VAA, CH, AW, OS and AH wrote the manuscript.

## Conflict of Interest Statement

The authors declare that the research was conducted in the absence of any commercial or financial relationships that could be construed as a potential conflict of interest.
